# Endovascular treatment of chronic aortic dissection in long term follow-up after cabrol procedure: a case report

**DOI:** 10.1186/1749-8090-8-S1-O297

**Published:** 2013-09-11

**Authors:** R Akchurin, T Imaev, P Lepilin, A Kolegaev, A Komlev, I Pokidkin

**Affiliations:** 1 Russian Cardiology Research Center, Moscow, Russia

## Background

Chronic and acute dissection of aorta is one of most common death causes in long-term follow up period in patients initially operated for aortic dissection. Although significant parts of cases are asymptomatic, such complications as residual or new-born dissection may result in neurological deficits, different vascular insufficiency, acute circulation collapse and death. Prompt treatment, therefore, should be employed in the management of this condition. To date, the standard treatment for recurrent aortic dissection is still surgical resection with distal end-to-end anastomosis. However, dissection repair and interposition grafting with artificial vessels are more invasive and time-consuming. The optimal treatment for such complicated cases should be relatively noninvasive, safe and free of significant complications, cost-effective, and incur less absence from usual daily activities. Endovascular stents have also been successfully used to treat dissections. Management of selected aneurysms by using stents has become more prevalent with the developing endoluminal technology.

## Case presentation

We report a case of an endovascular treatment of chronic aortic dissection, identified in long-term period after Cabrol procedure (19 years), where the patient was a 59 year-old man with a dissection in ascending part of the aorta with a distal fenestration in a level of left subclavian artery. He was treated with 130 x 40 E-XL (Jotec) endovascular aortic stent. The postoperative course of the patient was uneventful during the two-month follow-up.

## Conclusions

We show that there are significant early advantages with the endovascular management technique versus the conventional operation in the management of long-term complication of Cabrol aorta repair.

